# 16 Y/O Female with “Watermelon Stomach”?

**DOI:** 10.1155/2015/725341

**Published:** 2015-07-21

**Authors:** Amandeep Singh, Anwaar A. Khan, Robert Krall, Zafar K. Mirza

**Affiliations:** ^1^Department of Medicine, Olean General Hospital, Olean, NY, USA; ^2^Cleveland Clinic Foundation, Cleveland, OH, USA

## Abstract

*Background.* Gastric antral vascular ectasia (GAVE) also known as “watermelon stomach” (WS) is an uncommon cause of gastrointestinal (GI) blood loss. It typically presents in middle aged females. We are presenting a case of GAVE at an unusually early age with atypical symptoms. *Case.* A previously healthy 16 y/o Caucasian female presented to the ER with a one-month history of upper abdominal pain. Physical examination was benign except tenderness in the epigastric region. There were no significant findings on laboratory data. Upper endoscopy showed incidental findings of linear striae in the antrum indicative of GAVE but histology was equivocal. *Discussion.* GAVE is a poorly understood but treatable entity and an increasingly identifiable cause of chronic iron deficiency anemia or acute or occult upper GI bleeding. The pathophysiology of GAVE remains unclear. It is an endoscopic finding characterized by longitudinal columns of tortuous red ectatic vessels (watermelon stripes), pathognomonic for WS. Treatment options include endoscopic, pharmacologic, and surgical approaches. Failure to recognize GAVE can result in delayed treatment for years. Our patient with GAVE was unusually young and was diagnosed incidentally. Due to lack of anemia on laboratory examination we elected to monitor her clinically for any future development of anemia.

## 1. Introduction

Gastric antral vascular ectasia (GAVE) also known as “watermelon stomach” (WS) is the source of up to 4% of nonvariceal upper gastrointestinal (UGI) bleeding. Mostly, it presents as a cause of chronic iron deficiency anemia [1], but it can present with occult bleeding requiring transfusions or with acute gastrointestinal bleeding [2]. It typically presents in middle to old aged females with an average age of 73 years (range of 53–89 years) [3]. We are presenting a case of GAVE with atypical presentation and histology in a 16-year-old (y/o) white female with typical endoscopic findings.

## 2. Case

A 16 y/o obese white female presented to emergency department with one-month history of (h/o) off and on, upper abdominal pain, more in upper middle and right upper abdomen, 6/10, dull and achy in nature, constant, without any radiation, and associated with nausea and vomiting 2-3 times/day. It was not related to eating or drinking. There is no h/o acid reflux or difficulty swallowing. There is no change in appetite, weight, bowel pattern, or stool color. Each episode lasts 6-7 hours with minimal relief with heating pads and opioids. The patient reported no new medication, herbal products, or excessive use of over-the-counter pain medications.

She has no significant past medical or surgical history.

Neither was there a significant family history of autoimmune diseases, inflammatory bowel disease, or malignancy.

*Physical Examination*. Temperature was 98.2°F, pulse 72 beats/minute, and blood pressure 116/70 mm/Hg. She had no conjunctival pallor, scleral icterus, or lymphadenopathy. Cardiovascular and pulmonary examination was normal. Abdominal examination showed tenderness in right upper quadrant and midepigastric region and Murphy's sign was equivocal. The rest of the examination was normal.

*Laboratory Findings*. The findings are as follows: hemoglobin 14.4 g (normal 12.5–16), hematocrit 42.6 (normal 37–47), platelets 299.6 thousand/cmm (normal 150–450), white blood cells (WBC) 19.4 (normal 4–10.5), next morning 7.6, prothrombin time 11.4 seconds (normal 10.4–14.3), INR 1.02 (normal 0.9–1.1), aPTT 28.5 seconds (normal 0–40), total bilirubin 0.4 mg/dL (normal 0−1.2), AST 14 U/L (normal 5–34), ALT 16 U/L (normal 0–55), total protein 7.7 g/dL (normal 6–8), albumin 5 g/dL (normal 3.5–5), alpha1 AT 126 mg/dL (normal 83–199), cystic fibrosis screen being negative, lipase 11 units/L (normal 8–78), amylase 59 units/L (normal 5–65), TSH 3.93 mU/L (0.35–4.9), and negative rheumatoid factor, antinuclear antibodies, hepatitis B surface antigen (HBsAg), and HIV.

*Imaging*. Computerized tomography (CT) scan of abdomen/pelvis showed mild free fluid in the pelvis with small collapsed right ovarian cyst and with no other abnormality. Ultrasound abdomen showed cholelithiasis with gall bladder wall thickening of 4 mm with no stone or dilatation of common bile duct (CBD).

**Figure 1:**
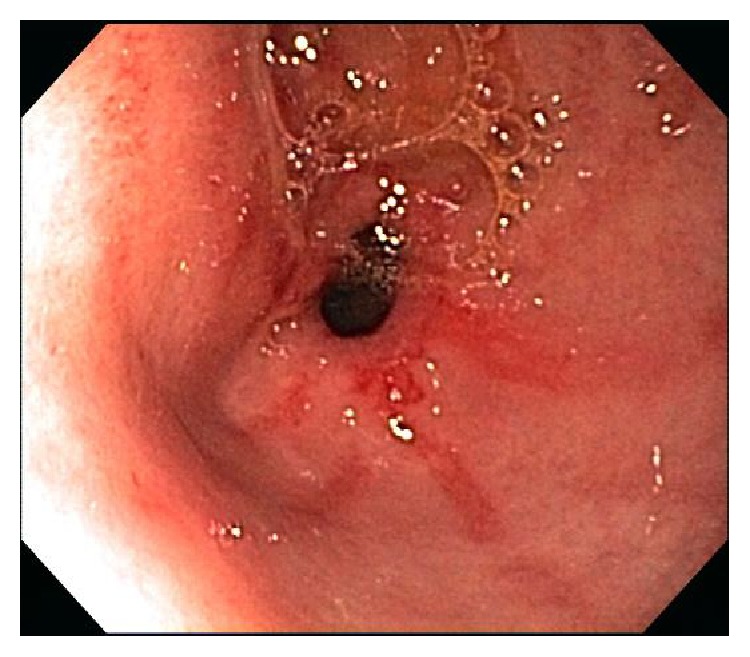
“Watermelon stomach” on upper endoscopy.

*Upper Endoscopy*. It showed watermelon stomach, linear stripes of antral telangiectasias (Figure 1) consistent with GAVE syndrome. CLO test (*Campylobacter*-like organism test) of antral biopsies was negative.

**Figure 2:**
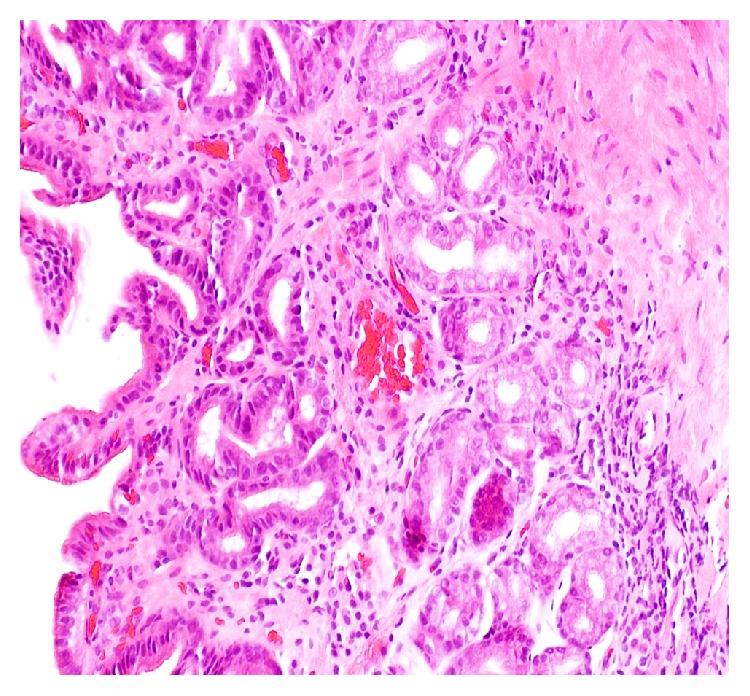
Inflammation at lamina propria and dilated capillary and venules.

*Histology*. Antral biopsy showed lamina propria normal thickness, mild chronic inflammation with few lymphocytes and plasma cells and dilated capillaries and venules. No fibrin thrombi were identified (Figure 2). The patient was started on pantoprazole and also had cholecystectomy but continued to have epigastric pain after 6 months without any occult bleeding or anemia.

## 3. Discussion

GAVE is a poorly understood but treatable entity and progressively increasingly identifiable cause of chronic iron deficiency anemia or acute or occult upper gastrointestinal bleeding. It is a challenging disease as the importance of this condition lies in its diagnosis; the failure to recognize GAVE results in delayed adequate treatment for years. It typically presents in middle to old aged females. In a single-center study of 45 patients with GAVE, 71% were female, with an average age of 73 years. In that study, approximately 89% of patients presented with transfusion dependent anemia [3]. There is only one case report in a 12 y/o Saudi girl with chronic gastritis mimicking watermelon stomach on endoscopy [4] and one case report where it presented as gastric outlet obstruction [5]. The first report of GAVE in the literature in 1953 was by Rider et al., who described the condition in an elderly female patient with chronic iron deficiency anemia [6]. An antrectomy specimen revealed “erosive atrophic gastritis with marked venocapillary ectasia” [3]. Jabbari et al., in 1984, described the characteristic endoscopic findings of “longitudinal antral folds … converging on the pylorus.” The endoscopic appearance resembled the stripes on a watermelon, and they coined the term “watermelon stomach” [7]. It has typical endoscopic findings. Histological pattern, although not pathognomic [8], is characterized by four different alterations: vascular ectasia of mucosal capillaries, focal thrombosis, spindle cell proliferation, and fibrinohyalinosis. “GAVE” score based on histological findings has high diagnostic accuracy (80%) to differentiate GAVE from portal hypertensive gastropathy, which may be present in patients with coexisting portal hypertension [9]. Generally, GAVE is associated with underlying chronic illnesses; cirrhosis of liver has been found in 30% of cases [10]. Among patients without cirrhosis, GAVE is usually associated with underlying autoimmune conditions.

Gostout et al. reported that 62% of patients had coexistent autoimmune connective tissue disorder, particularly Raynaud's phenomenon, which is reported in 31% of patients with GAVE [3]. Other underlying conditions include systemic sclerosis and CREST syndrome (calcinosis, Raynaud's syndrome, esophageal dysmotility, sclerodactyly, and telangiectasia) [3, 10–12]. The pathophysiology of GAVE remains unclear. Several theories have been proposed, including achlorhydria, hypergastrinemia, and low pepsinogen level. The pathogenesis of histological changes, most notably the lamina propria fibromuscular proliferation and vascular ectasia, is unclear. It has been suggested that failure of liver processing function may lead to a buildup of increased number of hormones with vasodilating properties which contribute to the pathogenesis of GAVE [13]. Watermelon stomach has pathognomonic endoscopic findings of the longitudinal antral folds containing visible columns of tortuous red ectatic vessels (watermelon stripes) and if missed can result in delay in treatment for many years. If diagnosed in time it could just be treated with iron supplementation in asymptomatic stable patient. Symptomatic anemic patients may need multiple transfusions to multiple endoscopic therapies with YAG laser or argon plasma or endoscopic band ligation. Treatment options for WS include pharmacologic, endoscopic, and surgical approaches. Endoscopic therapy, including contact and noncontact thermal ablations of the angiodysplastic lesions, is the mainstay of conservative therapy [14, 15]. Endoscopic interventions are usually recommended when patients become severely transfusion dependent or there is evidence of upper GI bleed. YAG laser can be used in patients with systemic sclerosis who develop chronic iron deficiency anemia. Argon plasma coagulation (APC) is an efficient and safe method of GAVE treatment in both cirrhotic and noncirrhotic patients in more than 80% of cases. Noncirrhotic patients required significantly more APC sessions to achieve a complete treatment. Recent studies showed that APC resulted in more frequent recurrences and needed multiple therapy sessions [16–18]. As compared to APC, endoscopic band ligation (EBL) is superior in the management of GAVE especially in patients with associated liver damage and portal hypertension. EBL required fewer treatment sessions for control of bleeding and caused the reduction in rehospitalization and transfusion requirements and allowed significant increase in hemoglobin values [19]. However, many patients fail endoscopic therapy and develop recurrent acute and chronic GI bleeding episodes. Surgical resection including antrectomy may be the only reliable method of achieving a cure and eliminating transfusion dependency [14]. Traditionally, surgery was used only as a last resort after patients failed prolonged medical and/or endoscopic therapy. However, recent studies recommend a more aggressive surgical approach in patients who failed a short trial of endoluminal therapy. One recent study showed that tranexamic acid may be a useful treatment for refractory bleeding due to GAVE in patients with cirrhosis [20]. Hemodialysis in patients with CKD associated with GAVE can reduce incidence of bleeding possibly by decreasing vascular congestion and decreasing mechanical stress. Restoration of platelet counts and endoscopic laser photocoagulation are the therapies of choice for ongoing bleeding in these patients [21]. Discontinuation of imatinib mesylate can benefit GAVE in patients with gastrointestinal stromal tumors (GIST) who are on imatinib [22].

It is not clear whether GAVE is just an endoscopic appearance which most commonly presents in anemic or bleeding patients or it has some other diagnostic significance as well. The association of GAVE with chronic inflammation seen in multiple conditions, which can cause chronic inflammatory changes in mucosa and later turn into typical histological features if present for long time, also needs to be elucidated. A question arises about the significance of repeat endoscopy in patients diagnosed with GAVE who are not anemic with no evidence of bleeding as endoscopy has its own risks.

## 4. Conclusion

GAVE is an atypical cause of chronic or acute anemia with typical endoscopic findings. It is usually presented in middle to old aged patients but can present in young age with atypical symptoms of right sided abdominal pain with nausea and vomiting. We need to keep this diagnosis in mind while performing endoscopies as the importance of this condition lies in early diagnosis and the failure to recognize GAVE can result in the delay of adequate treatment for a long time.
